# Is Male Hypogonadism a Risk Factor for Cancer Through Weakening of the Immune System?

**DOI:** 10.3390/ijms27146406

**Published:** 2026-07-18

**Authors:** Sandro La Vignera, Rosita A. Condorelli

**Affiliations:** Department of Clinical and Experimental Medicine, University of Catania, 95123 Catania, Italy

**Keywords:** hypogonadism, testosterone, immune system, neoplasms, inflammation, lymphocytes, cytokines, androgen replacement therapy, oxidative stress, cancer risk

## Abstract

Male hypogonadism is associated with metabolic and cardiovascular comorbidities, and emerging evidence implicates testosterone deficiency in immune dysregulation that may elevate cancer risk. To review current evidence on the relationship between male hypogonadism, immune function, and cancer risk, focusing on mechanisms linking testosterone deficiency to immune suppression and oncologic outcomes. PubMed/MEDLINE, Google Scholar, and SciSpace were systematically searched (through April 2026) using predefined search strings. After removal of duplicates (*n* = 1535 records screened), 156 full-text articles were assessed for eligibility; 20 studies met predefined inclusion criteria (comprising 4 experimental studies, 4 prospective/RCT studies, 7 observational studies, and 5 reviews used as secondary literature) and were included in a narrative synthesis. Testosterone deficiency was consistently associated with elevated IL-6, TNF-α, IL-1β, and CRP, impaired neutrophil maturation, and reduced NK-cell cytotoxicity. Androgen deprivation augmented thymic output and anti-tumor T cell responses in prostate cancer models, yet promoted chronic inflammation in other contexts. Epidemiologically, low testosterone correlated with increased colorectal cancer risk and poorer survival in advanced malignancies; the prostate cancer relationship followed a paradoxical saturation model. The immunological consequences of hypogonadism are context-dependent. Testosterone deficiency drives pro-inflammatory signaling that may promote carcinogenesis, while androgen-mediated immunosuppression can paradoxically impair anti-tumor surveillance. No simple linear relationship exists between hypogonadism and cancer risk via immune suppression. Prospective studies are needed to guide clinical decisions on testosterone replacement therapy in hypogonadal men.

## 1. Introduction

Male hypogonadism, defined as a clinical syndrome resulting from failure to produce physiological concentrations of testosterone and/or normal numbers of spermatozoa, affects an estimated 2–6% of men globally, with prevalence increasing substantially with age [1]. Beyond its well-established effects on reproductive function, sexual health, bone density, and muscle mass [2,3,4,5], testosterone has emerged as a critical regulator of immune system function [6,7]. This recognition has prompted an investigation into whether testosterone deficiency may influence cancer risk through alterations in immune surveillance and inflammatory processes.

The immune system plays a fundamental role in cancer prevention through continuous surveillance and elimination of transformed cells [8]. Immunodeficiency states, whether congenital or acquired, are associated with substantially increased cancer risk across multiple malignancy types [9]. Given testosterone’s documented immunomodulatory effects, it is biologically plausible that hypogonadism could compromise anti-tumor immunity and thereby increase cancer susceptibility [10].

However, the relationship between testosterone, immunity, and cancer is complex and potentially paradoxical. While testosterone deficiency is associated with increased systemic inflammation—a known cancer risk factor—testosterone itself can suppress certain aspects of adaptive immunity that are critical for tumor surveillance [11,12]. This apparent contradiction necessitates careful examination of the mechanisms through which testosterone influences different immune cell populations and inflammatory pathways, and how these effects may translate to cancer risk in hypogonadal men.

Epidemiological studies examining associations between testosterone levels and cancer incidence have yielded inconsistent results that vary substantially by cancer type [13,14]. The prostate cancer relationship is particularly complex, with evidence suggesting a paradoxical “saturation model” wherein low testosterone may associate with more aggressive disease rather than reduced risk [15]. For other cancer types, evidence is more limited and often confounded by the metabolic comorbidities that accompany hypogonadism.

Importantly, the temporal dynamics of testosterone deficiency may critically influence its immunological consequences: chronic deficiency, as observed in age-related hypogonadism, likely produces sustained but moderate immune alterations over years, whereas acute androgen ablation—as induced pharmacologically by androgen deprivation therapy—may trigger more rapid and profound immune changes with potentially distinct carcinogenic implications. This temporal distinction has not been adequately addressed in most available clinical studies and represents an important dimension of the mechanistic framework. This narrative review aims to synthesize current evidence on the relationship between male hypogonadism, immune function, and cancer risk. Specifically, we examine (1) testosterone’s mechanistic effects on different immune cell populations and inflammatory pathways; (2) immune alterations observed in hypogonadal men; (3) epidemiological associations between hypogonadism and cancer incidence across different malignancy types; and (4) potential mechanistic pathways linking testosterone deficiency, immune dysfunction, and carcinogenesis. We also discuss clinical implications and identify priorities for future research.

## 2. Results

### 2.1. Testosterone and Immune System Regulation: Mechanistic Evidence

Testosterone exerts complex, often opposing effects on different components of the immune system. At physiological concentrations, testosterone generally suppresses adaptive immunity while modulating innate immune responses in a context-dependent manner [6,7].

#### 2.1.1. Effects on T Lymphocytes

Testosterone demonstrates consistent immunosuppressive effects on T lymphocyte function across multiple experimental systems. In vitro studies show that physiological concentrations of testosterone inhibit T cell proliferation, reduce production of interleukin-2 (IL-2) and interferon-gamma (IFN-γ), and promote T cell apoptosis [16,17]. These effects are mediated through androgen receptor (AR) signaling, as demonstrated by studies using AR antagonists or AR-knockout models [16,18].

Kissick et al. [16] demonstrated in murine prostate cancer models that androgens inhibit Th1 differentiation and that AR-deficient mice show enhanced anti-tumor T cell responses. This primary experimental study provided direct evidence that androgen signaling suppresses T cell-mediated anti-tumor immunity. Complementing these findings, Sutherland et al. [19] showed in both castrated mice and men undergoing androgen deprivation therapy (ADT) for prostate cancer that castration reverses thymic involution and increases naive T cell output, indicating that physiological testosterone suppresses thymic function and T cell renewal.

Testosterone can also shift CD4+ T cell subsets away from pro-inflammatory Th1 and Th17 phenotypes toward regulatory T cells (Tregs) [16,20]. This shift may contribute to testosterone’s anti-inflammatory effects but could simultaneously impair anti-tumor immunity, as Tregs suppress effector T cell responses in the tumor microenvironment [21].

Clinical evidence from Page et al. [22] demonstrated that men undergoing medical castration with GnRH agonists experienced significant increases in both CD4+ and CD8+ T cell counts, confirming that testosterone suppresses T cell populations in humans. The magnitude of these changes and their functional consequences for immune surveillance remain areas of active investigation.

#### 2.1.2. Effects on B Lymphocytes

Testosterone suppresses B lymphocyte function and antibody production [23]. Experimental studies in rodents show that castration increases B cell numbers in bone marrow and spleen, while testosterone administration reduces B cell populations and immunoglobulin production [24,25]. Ponce et al. [26] reported that hypogonadal men exhibited elevated serum IgG, IgA, and IgM levels compared to eugonadal controls, with testosterone levels inversely correlated with immunoglobulin concentrations. This primary observational study provided human evidence that testosterone deficiency enhances humoral immunity.

The clinical significance of testosterone’s suppressive effects on B cells for cancer risk is unclear. While antibody responses can contribute to anti-tumor immunity in some contexts, B cells can also promote tumor progression through production of immunosuppressive cytokines and recruitment of regulatory immune cells [27].

#### 2.1.3. Effects on Natural Killer Cells

Natural killer (NK) cells are critical effectors of anti-tumor immunity, capable of recognizing and eliminating malignant cells without prior sensitization [28]. Testosterone reduces NK cell cytotoxic activity and decreases expression of activating receptors [29,30,31]. Page et al. [22] demonstrated that medical castration in men increased NK cell numbers, suggesting that physiological testosterone suppresses NK cell populations. Testosterone replacement therapy has been shown to reduce NK cell numbers and cytotoxic function [32], further supporting the conclusion that physiological testosterone may suppress NK cell-mediated tumor surveillance.

#### 2.1.4. Effects on Macrophages and Innate Immunity

Testosterone modulates macrophage polarization and function. Androgens promote M2 macrophage polarization—associated with anti-inflammatory, tissue-remodeling functions—over M1 polarization, which is associated with pro-inflammatory, anti-tumor activity [33,34]. Testosterone reduces macrophage production of TNF-α and IL-6 while promoting IL-10 production [35], consistent with a shift toward immunosuppressive macrophage phenotypes.

Neutrophil function is also modulated by testosterone. Testosterone promotes neutrophil maturation and enhances their antimicrobial functions but may also suppress their pro-inflammatory cytokine production [36,37]. The net effect of testosterone on innate immune defense against cancer is unclear.

#### 2.1.5. Effects on Thymic Function

The thymus undergoes involution with aging, a process accelerated by testosterone [19]. Castration reverses thymic involution and increases thymic output of naive T cells in both animal models and humans [19,38]. This testosterone-mediated thymic involution reduces the pool of naive T cells available for immune responses, potentially limiting the capacity to generate de novo anti-tumor T cell responses.

**Figure 1:**
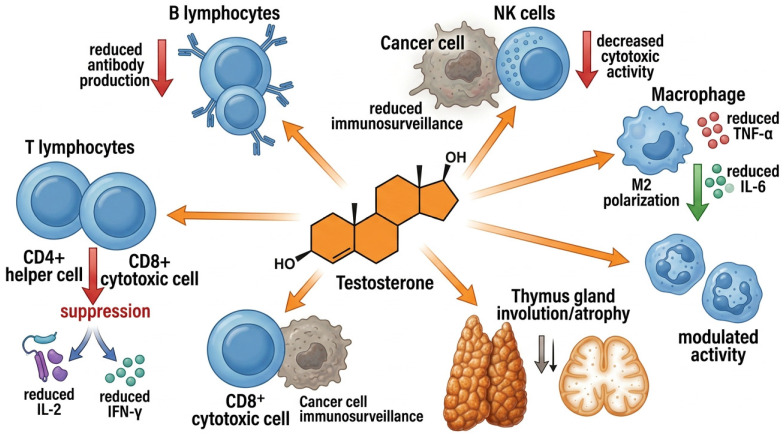
Schematic representation of the immunomodulatory effects of testosterone on immune cell populations and cancer immunosurveillance. Testosterone suppresses T lymphocyte (CD4+ and CD8+) proliferation and IL-2/IFN-γ production, decreases B lymphocyte antibody secretion, reduces NK cell cytotoxic activity, promotes M2 macrophage polarization with reduced TNF-α and IL-6, modulates neutrophil activity, and induces thymic involution. The net downstream effect is reduced immunosurveillance against cancer cells. Abbreviations: NK, natural killer; IL, interleukin; IFN-γ, interferon-gamma; TNF-α, tumor necrosis factor-alpha.

Figure 1 provides a schematic summary of testosterone’s immunomodulatory effects on immune cell populations and cancer immunosurveillance.

### 2.2. Immune Alterations in Hypogonadal Men

Multiple observational studies have documented altered immune profiles in hypogonadal men compared to eugonadal controls, though the pattern of alterations is complex and sometimes contradictory.

#### 2.2.1. Inflammatory Markers

Hypogonadal men consistently exhibit elevated circulating levels of pro-inflammatory markers. Maggio et al. [39] reported in a cohort of 1085 community-dwelling older men that low testosterone was inversely correlated with soluble IL-6 receptor, indicating enhanced IL-6 signaling in testosterone-deficient men. This association persisted after adjustment for age, body mass index, and comorbidities.

A systematic review by Mohamad et al. [40] synthesized evidence from multiple studies and concluded that an inverse relationship exists between testosterone and IL-6, TNF-α, and C-reactive protein (CRP) in men, with testosterone deficiency promoting systemic inflammation. Wang et al. [41] analyzed data from 7389 adult men in the National Health and Nutrition Examination Survey (NHANES) and found that a high systemic immune-inflammation index (SII)—a composite measure derived from platelet, neutrophil, and lymphocyte counts—was positively associated with testosterone deficiency prevalence. This large population-based study provided robust evidence that testosterone deficiency is associated with systemic inflammatory dysregulation.

Meta-analyses have estimated that each standard deviation decrease in testosterone is associated with approximately 0.1–0.2 standard deviation increases in inflammatory markers [42], though substantial heterogeneity exists across studies.

#### 2.2.2. Immune Cell Populations and Function

Evidence regarding immune cell populations in hypogonadal men is more limited and inconsistent. Ponce et al. [26] reported that hypogonadal men (*n* = 13) exhibited increased serum immunoglobulins (IgG, IgA, IgM) and elevated IL-2 and IL-4 levels compared to eugonadal controls (*n* = 10), suggesting enhanced cellular and humoral immunity. However, the small sample size limits generalizability.

Bianchi et al. [43] performed detailed immunophenotyping and found that hypogonadal men showed enhanced CD16+ dendritic cell activation, which was inversely correlated with testosterone levels. This suggests that testosterone deficiency may enhance antigen presentation capacity, potentially augmenting adaptive immune responses.

Studies in men with Klinefelter syndrome (47, XXY karyotype), who have congenital hypogonadism, have reported enhanced cellular and humoral immunity, with testosterone replacement therapy reducing IgG, IgA, IL-2, and IL-4 levels [44]. However, men with Klinefelter syndrome have multiple genetic and developmental differences beyond hypogonadism, limiting the ability to isolate testosterone’s effects.

#### 2.2.3. Effects of Testosterone Replacement Therapy on Immune Parameters

Testosterone replacement therapy (TRT) in hypogonadal men has been shown to reduce pro-inflammatory cytokines. The randomized controlled trial by Malkin et al. [45] demonstrated significant reductions in TNF-α, IL-1β, and IL-6 following TRT. Subsequent studies have confirmed these anti-inflammatory effects [46,47], though the magnitude of effect varies across studies and may depend on baseline inflammatory status, degree of testosterone deficiency, and TRT formulation and dosing.

### 2.3. Epidemiological Evidence: Hypogonadism and Cancer Incidence

Epidemiological studies examining associations between hypogonadism or testosterone levels and cancer incidence have yielded inconsistent results that vary substantially by cancer type.

#### 2.3.1. Prostate Cancer

The relationship between testosterone and prostate cancer is complex and paradoxical. Most large prospective studies have found no association or inverse associations between baseline testosterone levels and subsequent prostate cancer incidence [48,49]. A meta-analysis by the Endogenous Hormones and Prostate Cancer Collaborative Group [50] pooled data from 18 prospective studies including over 6000 prostate cancer cases and found no significant association between circulating testosterone levels and prostate cancer risk.

However, as synthesized in the review by Morgentaler and Traish [15], the relationship may follow a “saturation model” wherein prostate cancer growth is maximally stimulated at low testosterone concentrations, with further increases in testosterone having minimal additional effect. This model is consistent with clinical observations that men with low testosterone may present with more aggressive prostate cancer phenotypes [51,52]. Large-scale studies of testosterone replacement therapy have not demonstrated increased prostate cancer incidence [53,54], and current consensus guidelines indicate that TRT does not increase prostate cancer risk in appropriately selected and monitored men, though TRT is contraindicated in men with active prostate cancer [55]. It should be noted that epidemiological estimates in this section vary in the extent to which they are adjusted for key confounders such as BMI, insulin resistance, and systemic inflammation, and this variability should be considered when interpreting the data.

#### 2.3.2. Colorectal Cancer

Several studies have suggested that low testosterone may be associated with increased colorectal cancer risk. Watts et al. [56] analyzed data from 502,490 participants in the UK Biobank and found that lower testosterone levels were associated with modestly higher colorectal cancer incidence. Earlier studies have reported similar associations [55,57,58], though a meta-analysis found no overall association with substantial heterogeneity across studies [59]. The inconsistency across studies may reflect differences in population characteristics, testosterone measurement methodologies, and—critically—the degree of adjustment for metabolic confounders. Notably, colorectal cancer risk is strongly influenced by obesity, insulin resistance, and chronic inflammation, all of which are closely associated with hypogonadism; consequently, it is difficult to isolate the independent contribution of testosterone deficiency from these co-occurring metabolic factors. Whether reported epidemiological estimates are adjusted for BMI, insulin resistance, or systemic inflammation varies across studies and is not consistently reported. Mechanistically, an association between low testosterone and colorectal cancer could be mediated through increased systemic inflammation, metabolic dysfunction, altered gut microbiome composition [60], or through androgen receptor-mediated effects on colorectal epithelial cell proliferation and apoptosis. Overall, current evidence suggests a possible but inconsistent association between testosterone deficiency and colorectal cancer risk; prospective studies with comprehensive adjustment for metabolic confounders are needed to establish whether any relationship is independent of underlying metabolic comorbidities.

#### 2.3.3. Hematologic Malignancies

Limited evidence suggests a possible association between low testosterone and risk of certain hematologic malignancies, particularly non-Hodgkin lymphoma [61]. However, a large prospective study found no overall association between testosterone levels and hematologic malignancy risk [62], though statistical power to detect associations with specific lymphoma subtypes was limited. Men with Klinefelter syndrome exhibit increased risk for certain hematologic malignancies [44]; however, this population is characterized by multiple genetic and hormonal abnormalities beyond testosterone deficiency alone, and multiple factors likely contribute to this increased risk [63]. The immunological basis for a potential link between hypogonadism and hematologic malignancy could involve impaired NK-cell cytotoxicity and altered T cell homeostasis—both observed in testosterone-deficient states—which are critical for immune surveillance against lymphomas and leukemias. As with other cancer types, epidemiological estimates in this area vary in the degree of adjustment for metabolic cofactors, which limits interpretation. Overall, the evidence base for hematologic malignancies is substantially smaller and less consistent than for prostate or colorectal cancer; firm conclusions should not be drawn from the currently available data, and targeted prospective studies are warranted.

#### 2.3.4. Other Solid Tumors

Small studies have suggested possible associations between low testosterone and increased risk of lung cancer [64], gastric cancer [65], and hepatocellular carcinoma [66], but these findings require replication in larger, well-controlled studies. For most solid tumor types, insufficient evidence exists to draw conclusions about associations with hypogonadism.

#### 2.3.5. Cancer in Men Receiving Androgen Deprivation Therapy

Men receiving androgen deprivation therapy (ADT) for prostate cancer represent a unique population with iatrogenic severe hypogonadism. Some studies have suggested increased risk of second primary cancers, particularly bladder and colorectal cancers, in men on ADT [67,68]. However, these associations are difficult to interpret due to confounding by the underlying prostate cancer, increased medical surveillance, shared risk factors, and competing risks of mortality [69].

#### 2.3.6. Cancer Outcomes and Survival

Several studies have examined testosterone levels in men with established cancer. Almeida et al. [70] reported in a cross-sectional study of 119 male patients with advanced cancer that low testosterone was associated with elevated CRP, poor performance status, and shorter survival. Tvermosegaard et al. [71] found in a retrospective cohort of male survivors of childhood and testicular cancer that hypogonadism was linked to metabolic syndrome and elevated inflammatory markers, potentially increasing long-term health risks in cancer survivors.

### 2.4. Mechanistic Pathways Linking Testosterone, Immunity, and Cancer Risk

Integration of mechanistic and epidemiological evidence suggests several potential pathways through which testosterone deficiency might influence cancer risk, though the net effect appears to be complex and context-dependent.

#### 2.4.1. Chronic Inflammation and Carcinogenesis

Hypogonadism is consistently associated with elevated pro-inflammatory cytokines (IL-6, TNF-α, IL-1β) and acute phase reactants (CRP) [39,40,41]. Chronic inflammation promotes carcinogenesis through multiple mechanisms, as comprehensively reviewed by Grivennikov et al. [72] and Hanahan and Weinberg [73], including: DNA damage via reactive oxygen and nitrogen species; promotion of cell proliferation through NF-κB and STAT3 activation; inhibition of apoptosis; angiogenesis via VEGF upregulation; tissue invasion facilitated by matrix metalloproteinases [74]; and immunosuppression through recruitment of myeloid-derived suppressor cells and regulatory T cells.

#### 2.4.2. Altered Immune Surveillance

Testosterone’s suppressive effects on T lymphocytes and NK cells—key effectors of anti-tumor immunity—suggest that testosterone deficiency might paradoxically enhance immune surveillance [16,19,22]. Experimental studies in prostate cancer models demonstrate that androgen deprivation may augment anti-tumor T cell responses and can reduce tumor growth [16,75,76]. However, a critical caveat applies: these experimental findings derive predominantly from ADT and castration models, which induce profound, acute pharmacological androgen ablation. Such conditions are not directly comparable to the moderate, chronic testosterone deficiency that characterizes age-related hypogonadism. The immunological consequences of androgen depletion are likely dose- and duration-dependent, and conclusions derived from pharmacological castration models should not be uncritically extrapolated to the clinical setting of hypogonadism. Furthermore, the chronic inflammatory environment associated with hypogonadism may simultaneously promote immunosuppressive cell populations and create a tumor-permissive microenvironment [77], an effect mechanistically distinct from the acute immune activation observed with pharmacological castration.

#### 2.4.3. Metabolic Dysfunction and Indirect Effects

Hypogonadism is strongly associated with obesity, insulin resistance, metabolic syndrome, and type 2 diabetes [78,79]. These metabolic conditions are themselves established cancer risk factors [80,81]. Disentangling the direct immunological effects of testosterone deficiency from indirect effects mediated through metabolic dysfunction is challenging in observational studies [82]. Obesity and metabolic syndrome are associated with chronic low-grade inflammation, altered adipokine profiles, insulin resistance, and hormonal changes—all of which may influence cancer risk independently of testosterone’s direct immunological effects [83].

#### 2.4.4. Tissue-Specific and Context-Dependent Effects

The effects of testosterone on immunity and cancer risk likely vary substantially by tissue type, based on androgen receptor expression patterns, local immune microenvironments, and tissue-specific carcinogenic mechanisms [74,84]. Prostate tissue, which is uniquely androgen-dependent, exhibits a paradoxical relationship wherein low testosterone may be associated with more aggressive cancer despite testosterone’s role in normal prostate growth. In contrast, tissues without direct androgen dependence may be more influenced by testosterone’s systemic immunological and metabolic effects.

## 3. Discussion

### 3.1. Synthesis of Evidence: A Complex, Non-Linear Relationship

The relationship between male hypogonadism, immune function, and cancer risk is complex and does not support a simple linear model wherein testosterone deficiency weakens immunity and thereby increases cancer risk. Several key themes emerge from the synthesis of mechanistic, clinical, and epidemiological evidence.

First, testosterone is a potent immunomodulator with generally suppressive effects on adaptive immunity, particularly T lymphocyte and NK cell function [6,7,16,19,22]. These immunosuppressive effects are mediated through androgen receptor signaling and are observed consistently across experimental systems and species. The suppression of T cell and NK cell-mediated anti-tumor immunity by physiological testosterone suggests that testosterone deficiency might paradoxically enhance immune surveillance against malignant cells.

Second, hypogonadism is consistently associated with increased systemic inflammation, characterized by elevated pro-inflammatory cytokines (IL-6, TNF-α, IL-1β) and acute phase reactants (CRP) [39,40,41,45]. This chronic inflammatory state is a well-established cancer risk factor, promoting carcinogenesis through DNA damage, cell proliferation, inhibition of apoptosis, angiogenesis, and metastasis [72,73].

Third, despite these clear immunological alterations, epidemiological studies do not demonstrate consistent increases in overall cancer incidence in hypogonadal men [13,14,85,86]. The relationship varies substantially by cancer type: prostate cancer exhibits a paradoxical pattern wherein low testosterone may associate with more aggressive disease [15,51,52]; colorectal cancer shows some evidence of increased risk with low testosterone [56,57,58]; and for most other cancer types, evidence is insufficient or inconsistent.

This apparent paradox—clear immunological effects without consistent epidemiological associations—may be explained by the hypothesis that the immunosuppressive effects of testosterone on adaptive immunity partially offset the cancer-promoting effects of increased inflammation associated with testosterone deficiency [87,88]. This mechanistic hypothesis, however, remains unproven by direct experimental evidence and should be explicitly framed as a working hypothesis rather than a settled conclusion. To rigorously test this hypothesis, future research should include: (1) controlled in vitro studies comparing immune cell function across a physiological range of testosterone concentrations; (2) longitudinal in vivo studies linking immune phenotype to cancer outcomes in well-characterized hypogonadal populations; and (3) clinical studies examining whether testosterone replacement normalizes immune parameters in parallel with modulation of cancer risk.

An important alternative interpretation deserves explicit consideration: in many clinical settings, hypogonadism may not be a primary driver of cancer risk, but rather a biomarker of underlying metabolic or inflammatory conditions that independently contribute to cancer susceptibility. Obesity, insulin resistance, metabolic syndrome, and chronic systemic inflammation are well-established cancer risk factors and are among the most common causes of secondary testosterone deficiency [78,79,80,81]. In such settings, the observed epidemiological associations between low testosterone and cancer risk may largely represent confounding by the underlying metabolic disorder rather than a causal role for testosterone deficiency per se. This alternative pathway carries important clinical implications: addressing the underlying metabolic dysfunction may be more relevant to cancer risk reduction than testosterone supplementation in affected individuals. Future studies should explicitly account for this possibility by including comprehensive metabolic phenotyping and rigorous adjustment for BMI, insulin resistance, and systemic inflammatory markers.

### 3.2. Clinical Implications

Hypogonadism and Cancer Screening: Current evidence does not support considering hypogonadism as a major cancer risk factor that would warrant intensified cancer screening beyond standard age-appropriate recommendations [89,90]. While some associations have been observed for specific cancer types (particularly colorectal cancer), the magnitude of risk elevation is modest and inconsistent across studies.

Testosterone Replacement Therapy and Cancer Risk: Concerns about cancer risk should not be a primary factor in decisions regarding testosterone replacement therapy in appropriately selected hypogonadal men [91,92,93]. Large-scale studies and meta-analyses have not demonstrated increased overall cancer incidence with TRT [53,54,94]; however, this reassurance must be interpreted with caution, as most available studies have follow-up durations of less than five years [95]—a time horizon likely insufficient to detect long-term oncological consequences. Until longer-term surveillance data are available, clinical decisions regarding TRT should acknowledge this limitation. TRT remains contraindicated in men with active prostate or breast cancer [55,96].

Anti-Inflammatory Effects of TRT: The anti-inflammatory effects of TRT [45,46,47] may provide additional cardiovascular and metabolic benefits beyond the traditional indications for treatment [74,97]. However, whether these anti-inflammatory effects translate to reduced cancer risk over the long term remains uncertain.

Individualized Risk Assessment: Given the complex, context-dependent nature of testosterone’s immunological effects, cancer risk assessment in hypogonadal men should consider multiple factors including age, obesity, metabolic syndrome, family history, and specific cancer risk factors rather than focusing solely on testosterone status [98].

### 3.3. Limitations and Knowledge Gaps

Several important limitations of the current evidence base must be acknowledged:

1. **Study Design Limitations:** Most observational studies are cross-sectional [26,43,99], precluding causal inference. Longitudinal studies are needed.
2. **Short Follow-Up in Intervention Studies:** TRT studies typically have follow-up durations of less than 5 years [95,100], insufficient to assess long-term cancer outcomes.
3. **Testosterone Measurement Issues:** Single measurements may not reflect long-term exposure [101]; most studies measure total rather than free or bioavailable testosterone [102]; assay variability limits comparability [103].
4. Confounding by Age and Metabolic Factors: Hypogonadism is strongly associated with aging, obesity, and metabolic syndrome [78,79,97]; many studies have not adequately controlled for these confounders [104].
5. **Limited Mechanistic Data in Humans:** Most mechanistic studies were conducted in animal models [16,19,75,76]; translation to human physiology remains uncertain.
6. **Lack of Tumor Microenvironment Studies:** Most studies examined systemic immune parameters rather than the tumor microenvironment [105].
7. **Publication Bias:** Studies reporting positive associations may be preferentially published [106].
8. Cancer Type Heterogeneity: Pooling across cancer types obscures important biological heterogeneity [74,84].

### 3.4. Future Research Directions

To address these knowledge gaps, future research should focus on:

1. **Large-Scale, Long-Term Prospective Studies** with repeated testosterone measurements, detailed confounders assessment, and comprehensive cancer ascertainment [107].
2. **Mechanistic Studies in Human Tumor Microenvironments** examining immune cell populations and functional capacity within tumor tissues from hypogonadal versus eugonadal men [108].
3. **Repeated Hormone and Immune Measurements** in longitudinal studies to clarify temporal relationships [109].
4. **Investigation of Effect Modification** by age, obesity, metabolic status, and genetic polymorphisms in androgen receptor and immune genes [98].
5. **Tissue-Specific Mechanistic Studies** examining testosterone’s effects separately for different tissue types [110].
6. **Intervention Studies with Cancer Endpoints:** Randomized controlled trials of TRT with adequate power and follow-up to detect differences in cancer incidence [111].
7. **Biomarker Development:** Identification and validation of immune biomarkers that predict cancer risk in hypogonadal men [112].

## 4. Materials and Methods

### 4.1. Search Strategy and Information Sources

PubMed/MEDLINE, Google Scholar, and SciSpace were systematically searched from inception through April 2026 using the following predefined search strings. The detailed search strategy for each database is presented in Table 1.

### 4.2. Study Selection and Eligibility Criteria

**Inclusion criteria:**

1. Studies examining testosterone’s effects on immune cell function, immune cell populations, or inflammatory markers in preclinical (animal models) or clinical settings;
2. Studies assessing immune parameters in hypogonadal men compared to eugonadal controls;
3. Studies investigating associations between testosterone levels, hypogonadism status, and cancer incidence, mortality, or outcomes;
4. Studies evaluating effects of testosterone replacement therapy (TRT) on immune function or cancer risk;
5. Published in peer-reviewed journals;
6. Available in English.

**Exclusion criteria:**

7. Studies focused exclusively on female populations;
8. Studies examining only non-immunological mechanisms of testosterone action;
9. Case reports or small case series (*n* < 10) without mechanistic insights;
10. Conference abstracts without full-text availability;
11. Studies not available in English.

Both primary research studies (experimental, randomized controlled trials, cohort studies, cross-sectional studies) and relevant secondary literature (narrative reviews, systematic reviews, meta-analyses) were eligible for inclusion. Secondary literature was included to provide comprehensive coverage of established concepts and to contextualize primary findings, but was explicitly distinguished from primary evidence in the synthesis and interpretation.

**Figure 2:**
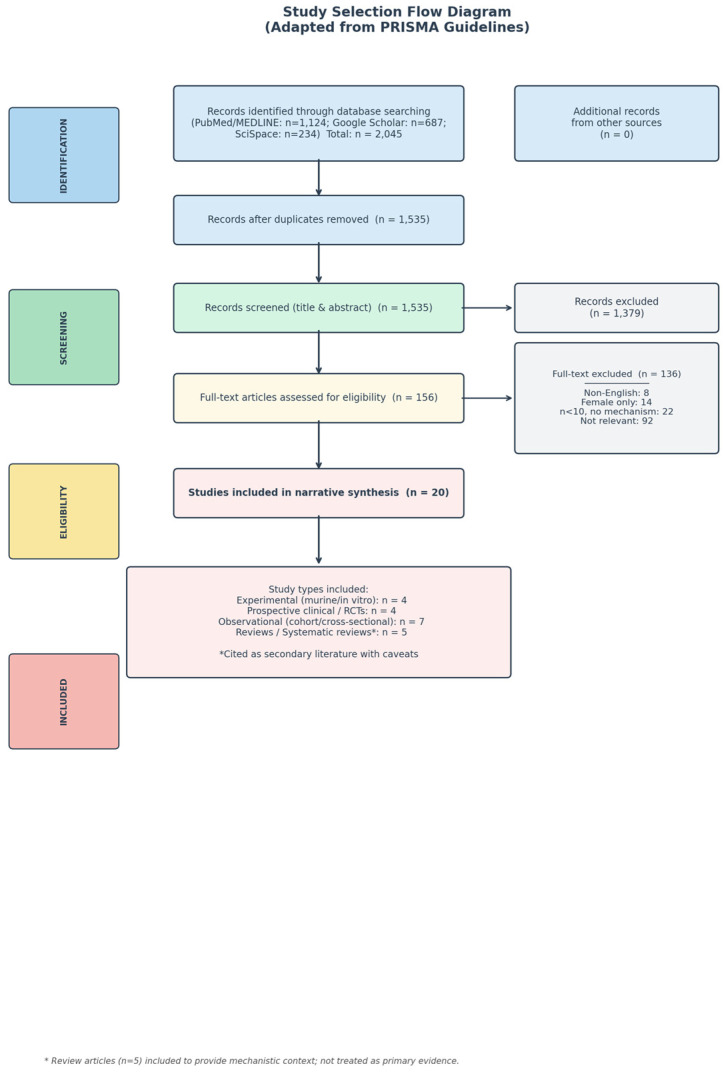
PRISMA-style flow diagram of the study selection process.

After screening, 20 studies met all inclusion criteria and were included in the final narrative synthesis. The study selection process is depicted in Figure 2.

### 4.3. Data Extraction and Synthesis

Data were extracted systematically from included studies using a standardized form. For each study, the following information was recorded: first author and year, study design, population/sample characteristics, testosterone measurement methods, immune outcomes assessed, cancer outcomes (if applicable), main findings, and study limitations.

Given the substantial heterogeneity in study designs (experimental vs. observational vs. interventional), populations (animal models, healthy men, hypogonadal men, cancer patients), immune outcomes (cytokines, immune cell counts, functional assays), and cancer types (prostate, colorectal, hematologic, other solid tumors), a narrative synthesis approach was deemed most appropriate rather than quantitative meta-analysis. Narrative synthesis allowed for comprehensive integration of diverse evidence types while preserving important contextual information about the complex, often opposing effects of testosterone on different immune compartments and cancer types.

Studies were grouped thematically according to: (1) testosterone’s effects on specific immune cell populations and inflammatory mediators; (2) immune alterations observed in hypogonadal men; (3) epidemiological associations between hypogonadism and cancer incidence/outcomes; and (4) mechanistic pathways linking immune dysfunction to cancer risk. Within each thematic area, findings from primary studies were synthesized first, followed by contextualization using relevant secondary literature.

### 4.4. Quality Assessment

Study quality was assessed using criteria appropriate to each study design, as no single quality assessment tool is suitable for the diverse study types included in this narrative review.

For **experimental (animal) studies**, quality was assessed based on: (1) use of appropriate control groups; (2) randomization and blinding where applicable; (3) adequate sample sizes with power calculations; (4) validated methods for testosterone manipulation and immune assessment; (5) control for confounding variables; and (6) reproducibility of findings.

For **randomized controlled trials (RCTs)**, quality was assessed using elements of the Cochrane Risk of Bias tool: (1) randomization method; (2) allocation concealment; (3) blinding of participants and personnel; (4) blinding of outcome assessment; (5) completeness of outcome data; (6) selective reporting; and (7) other sources of bias.

For **observational studies** (cohort, cross-sectional), quality was assessed based on: (1) representativeness of the study population; (2) adequate sample size; (3) validated methods for testosterone measurement (immunoassay vs. mass spectrometry); (4) measurement of total vs. free testosterone; (5) standardized immune assessment techniques; (6) cancer ascertainment methods (registry linkage, medical records, self-report); (7) length and completeness of follow-up (for cohort studies); (8) adjustment for key confounders (age, obesity, metabolic syndrome, smoking, comorbidities); and (9) appropriate statistical methods.

For **secondary literature** (reviews, systematic reviews, meta-analyses), quality was assessed based on: (1) comprehensiveness of search strategy; (2) explicit inclusion/exclusion criteria; (3) quality assessment of included studies; (4) appropriate synthesis methods; (5) assessment of publication bias (for meta-analyses); and (6) transparency regarding conflicts of interest and funding sources.

**Primary vs. Secondary Literature Distinction:**

Of the 20 included studies, 15 were classified as primary research (generating original data):

- Four experimental studies using animal models [16,19,75,76];
- Four clinical intervention or prospective studies [22,45,74,113];
- Seven observational studies (cohort, cross-sectional, case–control) [26,39,41,43,44,56,70].

Five studies were classified as secondary literature (synthesizing existing evidence):

- Three narrative reviews [15,72,73];
- Two systematic reviews [40,114].

Throughout the Results and Discussion sections, secondary literature is explicitly identified and cited with appropriate caveats. These sources are used to contextualize primary findings and establish theoretical frameworks, but are not treated as primary evidence for specific mechanistic or epidemiological claims.

An important methodological limitation of this review concerns its reliance on a relatively high proportion of secondary literature: five of the 20 included studies (25%) are classified as reviews (narrative or systematic), which may themselves reflect publication bias, selective citation, and heterogeneous methodological standards, introducing an additional layer of uncertainty. Furthermore, the limited number of primary experimental studies (*n* = 4) and randomized controlled trials (*n* = 4) constrains the strength of mechanistic and interventional inferences that can be drawn. These methodological constraints underscore the preliminary nature of the conclusions presented in this review and highlight the need for prospective, hypothesis-driven primary research to consolidate and extend current knowledge.

### 4.5. Handling of Study Heterogeneity

The included studies exhibited substantial heterogeneity across multiple dimensions, including study design, population, exposure characterization, and outcome measurement. Given this multidimensional heterogeneity, quantitative meta-analysis was not appropriate. Instead, narrative synthesis was chosen as the most suitable approach to preserve important contextual information, explore patterns across diverse evidence types, identify areas of convergence and divergence, and generate hypotheses regarding mechanisms and moderators of testosterone–immune–cancer relationships.

## 5. Conclusions

Current evidence does not support the hypothesis that male hypogonadism substantially increases cancer risk through weakening of the immune system. While testosterone clearly modulates immune function and hypogonadism is associated with altered immune profiles—particularly increased systemic inflammation—these immunological changes do not translate to consistently increased cancer incidence across epidemiological studies.

The relationship between testosterone, immunity, and cancer is complex and context-dependent. Testosterone exerts immunosuppressive effects on adaptive immunity (T cells, NK cells) that are critical for tumor surveillance, suggesting that testosterone deficiency might paradoxically enhance anti-tumor immunity. Simultaneously, testosterone deficiency is associated with chronic systemic inflammation, a well-established cancer risk factor. The net effect on cancer risk likely depends on the balance between these opposing mechanisms, which varies by cancer type, tissue microenvironment, and individual host factors.

From a clinical perspective, hypogonadism should not be considered a major cancer risk factor requiring intensified screening beyond standard age-appropriate recommendations. Concerns about cancer risk should not be a primary consideration in decisions regarding testosterone replacement therapy in appropriately selected hypogonadal men, though TRT remains contraindicated in men with active prostate or breast cancer. It is important to note, however, that this recommendation is based predominantly on studies with follow-up durations of less than five years [95], a time horizon that may be insufficient to detect long-term oncological consequences. Clinicians should communicate this uncertainty to patients and continue to monitor emerging long-term surveillance data as they become available.

Future research should focus on large-scale, long-term prospective studies with repeated hormone measurements and comprehensive immune profiling; mechanistic studies examining immune function in tumor microenvironments; investigation of effect modification by age, obesity, and metabolic status; and tissue-specific studies accounting for biological heterogeneity across cancer types.

## Figures and Tables

**Table 1:** Detailed search strategy and results by database.

Database	Search Terms	Date Range	Initial Results	After Deduplication
PubMed/MEDLINE	Search 1: (“male hypogonadism” OR “testosterone deficiency” OR “androgen deficiency”) AND (“immune system” OR “immune function” OR “immunosuppression”) AND (“cancer” OR “neoplasm” OR “malignancy”); Search 2: (“testosterone” OR “androgens”) AND (“T lymphocytes” OR “B lymphocytes” OR “NK cells” OR “macrophages” OR “cytokines” OR “inflammation”); Search 3: (“hypogonadism” OR “low testosterone”) AND (“cancer incidence” OR “cancer mortality” OR “prostate cancer” OR “colorectal cancer”)	Inception–April 2026	487	412
Google Scholar	Combined Boolean search: (hypogonadism OR “testosterone deficiency”) AND (immune OR immunity OR inflammation) AND (cancer OR neoplasm OR tumor)	Inception–April 2026	1240	856
SciSpace	Natural language query: “testosterone deficiency immune function cancer risk”; “hypogonadism inflammation malignancy”; “androgen deprivation immune response”	Inception–April 2026	318	267
Total	—	—	2045	1535

## Data Availability

No new data were created or analyzed in this study. Data sharing is not applicable to this article.

## Abbreviations

The following abbreviations are used in this manuscript:

**Abbreviation**	**Definition**
ADT	Androgen Deprivation Therapy
AR	Androgen Receptor
CRP	C-Reactive Protein
GnRH	Gonadotropin-Releasing Hormone
IFN-γ	Interferon-Gamma
Ig	Immunoglobulin
IL	Interleukin
NK	Natural Killer
RCT	Randomized Controlled Trial
ROS	Reactive Oxygen Species
SII	Systemic Immune-Inflammation Index
TGF-β	Transforming Growth Factor-Beta
TNF-α	Tumor Necrosis Factor-Alpha
Treg	Regulatory T Cell
TRT	Testosterone Replacement Therapy
VEGF	Vascular Endothelial Growth Factor
